# A Slower Age-Related Decline in Treatment Outcomes After the First Ovarian Stimulation for *in vitro Fertilization* in Women With Polycystic Ovary Syndrome

**DOI:** 10.3389/fendo.2019.00834

**Published:** 2019-12-05

**Authors:** Jing Li, Xiaocong Liu, Linli Hu, Fuli Zhang, Fang Wang, Huijuan Kong, Shanjun Dai, Yihong Guo

**Affiliations:** ^1^Center for Reproductive Medicine, The First Affiliated Hospital of Zhengzhou University, Zhengzhou, China; ^2^Henan Key Laboratory of Reproduction and Genetics, The First Affiliated Hospital of Zhengzhou University, Zhengzhou, China

**Keywords:** PCOS, advanced age, live birth rate, cumulative live birth rate, *in vitro fertilization*, BMI

## Abstract

**Background:** Polycystic ovary syndrome (PCOS) patients have a better ovarian reserve and age-related improvement in endocrine disturbances than non-PCOS patients. The effects of age on *in vitro fertilization* (IVF) treatment outcomes associated with cumulative live birth rate (CLBR) remain unclear.

**Objectives:** To study the effect of age on CLBR after the first ovarian stimulation in IVF in PCOS patients.

**Method:** This retrospective cohort study included 3,502 PCOS patients and 18,596 patients with tubal factor infertility, who underwent their first IVF cycles and subsequent frozen embryo transfer (ET) attempts. The primary outcome was CLBR associated with a single stimulation cycle and secondary outcomes included the implantation rate, clinical pregnancy rate, live birth rate (LBR), large for gestational age (LGA) rate, small for gestational age (SGA) rate, and preterm birth (PTB) rate of fresh ET cycles.

**Results:** PCOS patients over 40 years had a higher implantation rate (27.8 vs. 15.7%, *P* < 0.05), clinical pregnancy rate (51.4 vs. 26.1%, *P* < 0.05), LBR (42.3 vs. 18.2%, *P* < 0.05), and CLBR (50.0 vs. 21.5%, *P* < 0.05) than non-PCOS patients over 40 years. These rates were comparable between PCOS patients aged 35 to 40 years and those aged over 40 years (*P* = 0.263, 0.385, and 0.112, respectively). The changes in the implantation rate, clinical pregnancy rate, and CLBR by age were slower for PCOS patients than for non-PCOS patients (all *P* < 0.05). Among PCOS patients less than 35 years, BMI was negatively associated with CLBR [aOR: 0.961 (0.939–0.985); *P* < 0.05]; however, among PCOS patients over 35 years, instead of BMI (*P* = 0.353), age [aOR: 0.891 (0.803–0.990); *P* < 0.05] and the number of oocytes retrieved [aOR: 1.093 (1.002–1.078); *P* < 0.05] were significantly associated with CLBR. No significant differences in LGA, LGA, or PTB were detected between PCOS and non-PCOS patients over 35 years (all *P* > 0.05).

**Conclusions:** The declines in treatment outcomes with age are slower for PCOS patients than for non-PCOS patients. For patients over 40 years, PCOS patients have reproductive advantages over non-PCOS patients. In contrast to younger PCOS patients (<35 years), older PCOS patients (≥35 years) may benefit less from taking time to lose weight before IVF treatment, and the immediate initiation of assisted reproductive treatment is essential.

## Introduction

Polycystic ovary syndrome (PCOS) is an endocrine disorder characterized by irregular menses, elevated androgen levels, hirsutism, insulin resistance, and polycystic ovaries, and it accounts for 18–25% of infertility among couples (1). Age is a key factor affecting female fertility, because with increasing age, the reproductive capacity of a woman declines due to reduced ovarian reserve, oocyte quality, and the increased prevalence of embryonic aneuploidy (2–4). An almost linear decrease in fertility is observed in women after the age of 35 years, but the fertility trend of women with PCOS may be an exception (2, 5). Early studies have suggested that the serum anti-Mullerian hormone (AMH) level and antral follicle count (AFC), as surrogate markers of the ovarian reserve, decline with age in normo-ovulatory women, while this kind of age-related decrease is slower in PCOS patients, and levels are higher in women with PCOS than in normo-ovulatory women of the same age (6, 7). Other studies have further shown that a small portion of women experience improvements in the symptoms and signs of PCOS after the age of 40 years, such as the improvement of irregular menses and hirsutism and a decline in androgen levels (8).

The age-related improvement of endocrine disturbance and relatively good ovarian reserve does not indicate that PCOS patients will acquire reproductive superiority over normo-ovulatory women, as other factors that affect female fertility still cannot be ignored. For example, insulin resistance, as a common feature of PCOS that has been suggested to show a specific ovarian ultrasound pattern, may persist or even worsen in PCOS patients with advancing age (9, 10). Some extra- and/or intra-ovarian factors may negatively affect oocyte quality and embryonic developmental potential and may thus result in adverse pregnancy outcomes among patients with PCOS (11).

Further investigations are essential in this field to elucidate the reproductive advantages and detrimental factors of women with PCOS to enable rational birth plans. Previous studies have mainly focused on the pregnancy outcomes of fresh embryo transfer cycles, which cannot provide comprehensive information on the likelihood of ultimate success in *in vitro fertilization* (IVF) success (12, 13). Evaluating the cumulative live birth rate (CLBR) is actually a better method for estimating the effects of age on the treatment outcomes of PCOS patients (14, 15). We aimed to evaluate changes in the age-related reproductive characteristics of women with PCOS and analyze the critical parameters influencing the treatment outcomes associated with a single stimulation cycle.

## Materials and Methods

### Patients

This retrospective study was based on the Clinical Reproductive Medicine Management System/Electronic Medical Record Cohort Database (CCRM/EMRCD) at the Reproductive Medical Center, First Affiliated Hospital of Zhengzhou University, and the Henan Province Key Laboratory for Reproduction and Genetics. Patients who had endometriosis, genetic diseases, preimplantation genetic testing (PGT), ovarian surgery, uterine malformations, or other endocrine-metabolic disorders, such as thyroid dysfunction, congenital adrenal disease, Cushing's syndrome, or hyperprolactinemia, were excluded. Among 22,098 patients, 3,502 were diagnosed with PCOS, and the remaining 18,596 were diagnosed with infertility due to tubal factors. The diagnosis of PCOS by clinicians in the electronic medical record was based on the 2003 Rotterdam consensus (16), in which the presence of at least two of the following three features was indicated: oligo-ovulation or anovulation; clinical and/or biochemical hyperandrogenism; and polycystic ovaries in a transvaginal ultrasound exam.

Patients underwent IVF/ET using a standard long protocol and were followed up for at least 2 years until they either delivered at least one live baby or had used up all their embryos in subsequent frozen embryo transfer (FET) cycles. Data on pregnancy outcomes and fetal/neonatal outcomes were recorded.

### Dataset

The 22,098 cases were stratified into four groups based on patient age and diagnosis: younger group (age <35 years): group A (non-PCOS, *n* = 14,038) and group B (PCOS, *n* = 3,144); and older group (age ≥ 35 years): group C (non-PCOS, *n* = 4,558) and group D (PCOS, *n* = 358). The women aged ≥35 years were further divided into four subgroups: group C1 (non-PCOS, 35–40 years, *n* = 3,311); group D1 (PCOS, 35–40 years, *n* = 316); group C2 (non-PCOS, ≥40 years, *n* = 1,247), and group D2 (PCOS, ≥40 years, *n* = 42).

### IVF Protocol

Ovarian stimulation was performed with a standard long protocol. Daily injections of Gonal-F (follicle-stimulating hormone: Serono, Switzerland) were administered after complete suppression of ovulation using injections of decapeptyl (gonadotropin-releasing hormone agonist: Ferring, Germany) or diphereline (Ipsen, France). The initial dose of Gn ranged from 112.5 to 300 IU according to the body mass index (BMI) and ovarian function of the patients. Follicular development was monitored via transvaginal ultrasound examination. The gonadotropin (Gn) dose was adjusted based on follicle size and endocrine levels. Human chorionic gonadotrophin (hCG, Livzon, China) was injected when the largest follicle was more than 20 mm in diameter, and follicles > 16 mm accounted for more than 2/3 of the total follicles. In addition, the trigger criterion was applied flexibly by taking into account the peak E2 levels and progesterone levels. Thirty-seven hours after injection, oocyte retrieval was performed under ultrasonic guidance followed by IVF.

On the day of oocyte retrieval and ET, every woman was assessed for her risk of ovarian hyperstimulation syndrome (OHSS). ET was canceled for women with significant symptoms of OHSS. Criteria for the diagnosis of OHSS ranged from nausea, vomiting, abdominal discomfort, and ovarian enlargement (>5 cm) to pleural or peritoneal effusion, oliguria, abnormal liver function, renal failure, and thrombosis (17). Embryos were frozen at the cleavage stage or cultured to the blastocyst stage before vitrification according to morphologic criteria for anticipated transplantation in subsequent cryopreservation cycles. One to three embryos were transferred on day 2 or day 3 after follicle aspiration or in the subsequent FET cycles, natural cycle or under artificial preparation based on the available number of embryos, age of the patient, and whether the subject had a uterine scar, cervical insufficiency, a uterine malformation, or abnormal pelvic morphology, etc. All study protocols were approved by the Ethics Committee of the First Affiliated Hospital of Zhengzhou University (Scientific Research-2016-011).

### Outcomes

Fourteen or 18 days after ET, quantitative determination of the hCG content in blood was used to diagnose early pregnancy, and 35 or 45 days after ET, ultrasound was performed to detect the presence of an intrauterine gestational sac and positive cardiac pulsations, indicating clinical pregnancy. Multiple pregnancy was defined as the presence of more than one intrauterine gestational sac. Miscarriage was defined as spontaneous pregnancy loss at up to 24 weeks of gestation. The live birth was defined as at least one live delivery after 24 weeks of gestation. The CLBR was defined as the delivery of at least one live birth in the fresh or in the subsequent FET cycles, and only the first live birth event was considered in the analysis. The implantation rate was defined as the number of gestational sacs/number of transferred embryos. Preterm birth (PTB) was defined as the birth of a baby before 37 weeks of gestation. Small for gestational age (SGA) denoted a birth weight less than the tenth percentile for the average birth weight of the same gestational age. Large for gestational age (LGA) denoted a birth weight above the 90th percentile of the average birth weight for the same gestational age. A telephone follow-up assessment was conducted to record data on miscarriage, gestational age, and neonatal weight.

### Statistical Analysis

SPSS 17.0 (IBM) was used for statistical analyses. Continuous variables are presented as the mean ± SD, and categorical variables are presented as frequencies and percentages. Comparisons among groups were performed with one-way ANOVA followed and the Bonferroni test, chi-square test, and Fisher's exact test. To assess age-related change in reproductive characteristics, multiple regression analyses and single-factor logistic regression were performed in the PCOS and control groups separately, using age, BMI, total Gn dose, and the number of retrieved oocytes as independent variables. Adjusted odds ratios (aORs) with 95% confidence intervals (CIs) were estimated. All tests were two-sided, and statistical significance was defined as *P* < 0.05.

## Results

**Figure 1:**
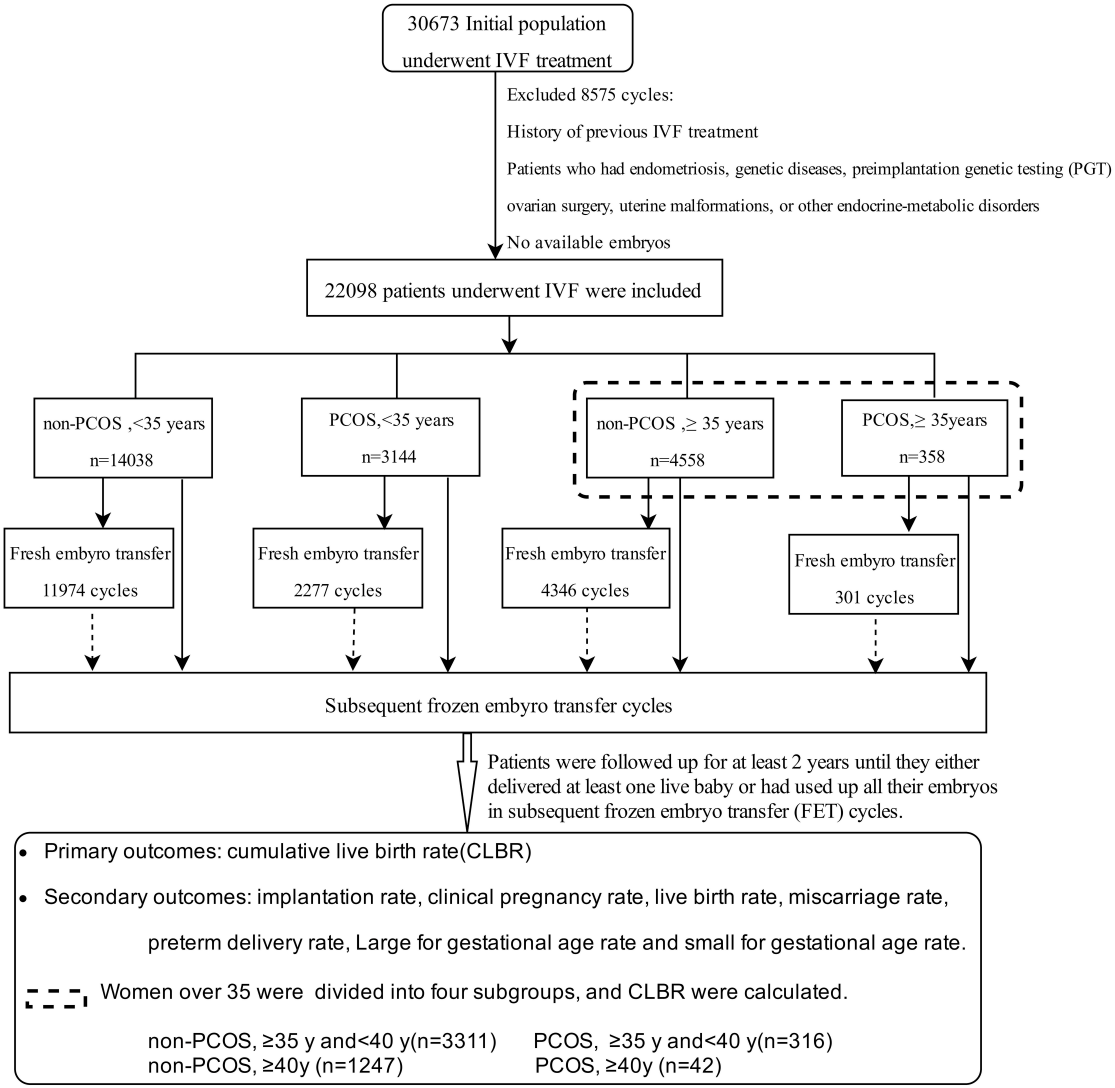
Flow chart of patients enrolled, the grouping, and the main treatment outcomes that were calculated in the study.

A total of 22,098 patients undergoing their first IVF-embryo transfer (ET) attempts between January 2010 and July 2015 were included in this study, and all patients were followed up for at least 2 years (Figure 1). Differences in the implantation rate, clinical pregnancy rate, miscarriage rate, live birth rate (LBR), preterm delivery rate, LGA rate, SGA rate, and CLBR were compared among these four groups. Age-related changes in treatment outcomes among women with or without PCOS were analyzed.

### General Data

The primary infertility rate was significantly higher in the PCOS group than in the age-matched non-PCOS group (group D vs. C, group B vs. A; all *P* < 0.05). There were significant differences in age, BMI, LH levels, T levels, total Gn dosage, and peak E2 levels among group A, B, C, and D (all *P* < 0.05). Significant differences were observed in the number of retrieved oocytes and available embryos among the four groups (all *P* < 0.05), except between groups A and D (*P* > 0.05; Table 1).

**Table 1:** Basic clinical characteristics of the PCOS and non-PCOS patients in the different age groups.

**Variable**	**Non-PCOS, <35 years (Group A)**	**PCOS, <35 years (Group B)**	**Non-PCOS, ≥35 years (Group C)**	**PCOS, ≥35 years (Group D)**
Total number of IVF cycles	14,038	3,144	4,558	358
Primary infertility, %^a,b,c,d^	61.6 (8,645/14,038)	68.6 (2,156/3,144)	26.9 (1,225/4,558)	32.7 (117/358)
Secondary infertility, %^a,b,c,d^	38.4 (5,393/14,038)	31.4 (988/3,144)	73.1 (3,333/4,558)	67.3 (241/358)
Age (y)^a,c,d^	28.75 ± 3.28	28.17 ± 3.14	38.12 ± 2.78	36.99 ± 2.20
BMI (kg/m^2^)^a,b,c,d^	22.04 ± 2.99	23.28 ± 3.42	23.01 ± 2.84	24.49 ± 3.39
Baseline LH (IU/L)^a,b,d^	5.10 ± 2.18	9.37 ± 7.94	4.87 ± 2.06	8.17 ± 8.85
Baseline FSH (IU/L)	7.08 ± 2.37	6.23 ± 2.03	7.75 ± 3.05	7.01 ± 5.53
Baseline T (ng/mL)^a,b,d^	0.70 ± 6.24	1.13 ± 4.26	0.46 ± 2.64	0.85 ± 4.51
Gn dosage (IU)^a,b,c,d^	2055.73 ± 840.56	1855.88 ± 869.90	2960.86 ± 944.93	2775.19 ± 1116.56
Peak E2^a,b,c,d^	4928.57 ± 2898.60	5471.69 ± 3278.92	3858.09 ± 2509.47	4634.43 ± 2953.68
No. of retrieved oocytes ^a,b,c,d^	11.76 ± 5.93	14.55 ± 7.20	8.52 ± 5.27	12.20 ± 6.69
No. of available embryos ^a,b,c,d^	5.55 ± 3.32	6.57 ± 3.90	4.07 ± 2.65	5.84 ± 3.86
No. of embryos transferred ^c,d^	2.03 ± 0.29	2.04 ± 0.28	2.15 ± 0.49	2.18 ± 0.49
OHSS cycles, % ^a,b,c^	12.8 (1,800/14,038)	25.8 (812/3,144)	3.3 (149/4,558)	13.4 (48/358)
Fresh embryo ET cycles, %^a,b,c,d^	85.3 (11,974/14,038)	72.4 (2,277/3,144)	95.3 (4,346/4,558)	84.1 (301/358)
ET with a single embryo, %^a,b,c^	4.1 (490/11,974)	3.1 (70/2,277)	10.8 (468/4,346)	4.3 (13/301)
ET with two embryos, %^a,c,d^	91.4 (10,940/11,974)	92.9 (2,115/2,277)	74.1 (3,222/4,346)	77.7 (234/301)
ET with three embryos, %^c,d^	4.6 (545/11,974)	4.0 (92/2,277)	15.1 (656/4,346)	17.9 (54/301)
Implantation rate, %^b,c,d^	46.7 (11,207/24,005)	44.3 (2,029/4,576)	28.8 (2,560/8,880)	36.4 (234/643)
No. of frozen day 2-3 embryos^a,b,c,d^	4.90 ± 3.28	5.56 ± 3.89	3.96 ± 2.74	4.90 ± 4.27
No. of frozen day 5-6 embryos^a,c,d^	2.58 ± 1.90	2.85 ± 2.01	2.05 ± 1.53	2.01 ± 1.58

*LH, Luteinizing hormone; FSH, Follicle-stimulating hormone; T, Testosterone; E2, Estradiol; Gn, gonadotropin; OHSS, Ovarian hyperstimulation syndrome; ET, Embryo transfer. Data are presented as the mean ± SD. “a”, “b”, “c,” and “d” mean that significant differences were detected between groups A and B, C, and D, A and C, and B and D, respectively*.

Univariate analyses showed that age, BMI, baseline LH levels, peak E2 levels on the day of hCG injection, the number of retrieved oocytes, and the number of embryos transferred were correlated with the pregnancy rate, LBR, singleton pregnancy rate and multiple pregnancy rate (all *P* < 0.05) and that age and BMI were correlated with the miscarriage rate and preterm delivery rate (all *P* < 0.05). In addition, age, BMI, and gestational age were correlated with the LGA and SGA rate (all *P* < 0.05). Age, BMI, total Gn, and number of oocytes retrieved were shown to be correlated with the CLBR among the four groups (all *P* < 0.05). The variables correlated with the treatment outcomes were subjected to multivariate logistic regression.

### Pregnancy Outcomes of Fresh Embryo Transfer Cycles

The OHSS rate was significantly higher in the PCOS group than in the age-matched non-PCOS group (group D vs. C, group B vs. A; all *P* < 0.05). For PCOS patients, no significant in OHSS rate was detected between the younger and older group (group B vs. D; *P* > 0.05). The double-embryo transfer rate was significantly higher in the younger than in the older groups (group A vs. C, group B vs. D; all *P* < 0.05). In contrast, a significantly higher one- or three-embryo transfer rate was observed in older groups than in their younger counterparts (group C vs. A, group D vs. B; all *P* < 0.05). For women at an advanced age, the PCOS group showed a higher implantation rate than did the non-PCOS group (group D vs. C; *P* < 0.05; Table 1). A significantly higher implantation rate was observed in both the group of PCOS patients aged 35–40 years and the group of PCOS patients over 40 years old than in the group of age-matched non-PCOS patients (all *P* < 0.05; **Table 4**).

There was no significant difference between group B and group A in clinical pregnancy rate (*P* > 0.05). Group B exhibited a higher miscarriage rate [aOR: 1.386 (1.164–1.650); *P* < 0.05] and a lower LBR [aOR: 0.864 (0.777–0.960); *P* < 0.05] than group A. Group D exhibited a higher clinical pregnancy rate [aOR: 1.307 (1.012–1.688); *P* < 0.05] and LBR [aOR: 1.297 (1.015–1.657); *P* < 0.05] than group C, while no difference was observed in the miscarriage rate (*P* > 0.05) between the two groups (Table 2). Among non-PCOS patients, group C had a higher miscarriage rate [aOR:1.409 (1.231–1.614); *P* < 0.05] than group A, while among PCOS patients, the miscarriage rates were comparable between the different age groups (all *P* > 0.05; Table 3). Additionally, among the subgroups (Groups C1, C2, D1, and D2), no significant differences in the miscarriage rate (*P* = 0.389) were detected (Table 4).

**Table 2:** Differences in pregnancy outcomes between women with or without PCOS.

**Variable**	**<35 years**			**≥35 years**		
	**Non-PCOS (Group A)**	**PCOS (Group B)**	***P*-value**	**Non-PCOS (Group C)**	**PCOS (Group D)**	***P*-value**
Clinical pregnancy rate, %	68.4 (8,191/11,974)	65.5 (1,491/2,277)		47.0 (2,044/4,346)	59.1 (178/301)	
OR (95% CI)	REF	0.876 (0.797–0.963)	0.006	REF	1.630 (1.285–2.067)	0.000
aOR (95% CI)	REF	0.931 (0.833–1.041)	0.209	REF	1.307 (1.012–1.688)	0.040
Miscarriage rate, %	5.7 (684/11,974)	7.9 (181/2,277)		8.0 (349/4,346)	9.0 (27/301)	
OR (95% CI)	REF	1.428 (1.204–1.693)	0.000	REF	1.129 (0.749–1.701)	0.563
aOR (95% CI)	REF	1.386 (1.164–1.650)	0.000	REF	1.129 (0.749–1.701)	0.563
Live birth rate, %	61.5 (7,362/11,974)	56.2 (1,280/2,277)		38.4 (1,668/4,346)	49.2 (148/301)	
OR (95% CI)	REF	0.804 (0.735–0.881)	0.000	REF	1.553 (1.229–1.963)	0.000
aOR (95% CI)	REF	0.864 (0.777–0.960)	0.007	REF	1.297 (1.015–1.657)	0.038
Singleton pregnancy rate, %	39.5 (4,728/11,974)	35.6 (811/2,277)		29.2 (1,267/4,346)	33.9 (102/301)	
OR (95% CI)	REF	0.848 (0.772–0.931)	0.001	REF	1.246 (0.973–1.585)	0.082
aOR (95% CI)	REF	0.877 (0.799–0.964)	0.006	REF	1.038 (0.807–1.336)	0.772
Preterm delivery, %	5.5 (269/4,728)	8.2 (67/811)		7.8 (99/1,267)	8.8 (9/102)	
OR (95% CI)	REF	1.531 (1.162–2.017)	0.002	REF	1.145 (0.580–2.258)	0.697
aOR (95% CI)	REF	1.419 (1.067–1.888)	0.016	REF	0.993 (0.498–1.979)	0.984
Large for gestational age, %	27.4 (1,295/4,728)	31.3 (254/811)		31.2 (395/1,267)	33.3 (34/102)	
OR (95% CI)	REF	1.166 (0.993–1.369)	0.061	REF	1.019 (0.670–1.549)	0.931
aOR (95% CI)	REF	1.166 (0.993–1.307)	0.061	REF	1.037 (0.681–1.578)	0.865
Small for gestational age, %	3.9 (186/4,728)	3.3 (27/811)		4.9 (62/1,267)	0.9 (1/102)	
OR (95% CI)	REF	0.820 (0.544–1.236)	0.343	REF	0.183 (0.025–1.329)	0.093
aOR (95% CI)	REF	0.821 (0.544–1.238)	0.346	REF	0.169 (0.023–1.232)	0.079
Multiple pregnancy rate, %	22.0 (2,634/11,974)	20.6 (469/2,277)		9.2 (401/4,346)	15.3 (46/301)	
OR (95% CI)	REF	0.920 (0.824–1.027)	0.138	REF	1.775 (1.275–2.469)	0.001
aOR (95% CI)	REF	0.891 (0.790–1.006)	0.063	REF	1.413 (1.006–1.983)	0.046
Preterm delivery,%	34.3 (1,807/5,268)	39.6 (371/938)		39.8 (319/802)	45.0 (41/92)	
OR (95% CI)	REF	1.254 (1.021–1.541)	0.031	REF	1.239 (0.642–2.392)	0.524
aOR (95% CI)	REF	1.171 (0.950–1.444)	0.138	REF	1.042 (0.527–2.060)	0.905
Large for gestational age, %	3.7 (196/5,268)	3.9 (37/938)		4.2 (34/802)	0 (0/92)	
OR (95% CI)	REF	1.090 (0.761–1.561)	0.638	REF	–	0.997
aOR (95% CI)	REF	1.096 (0.765–1.569)	0.618	REF	–	0.997
Small for gestational age, %	27.8 (1,252/5,268)	23.1 (217/938)		21.6 (173/802)	23.9 (22/92)	
OR (95% CI)	REF	0.996 (0.843–1.177)	0.966	REF	1.145 (0.679–1.930)	0.613
aOR (95% CI)	REF	0.990 (0.838–1.171)	0.910	REF	1.187 (0.702–1.171)	0.523
Cumulative live birth rate,%	72.6 (10,186/14,038)	69.3 (2,180/3,144)		45.3 (2,064/4,558)	60.6 (217/358)	
OR (95% CI)	REF	0.855 (0.783–0.928)	0.005	REF	1.857 (1.491–2.341)	0.000
aOR (95% CI)	REF	0.755 (0.710–0.846)	0.000	REF	1.399 (1.101–1.779)	0.006

*OR, odds ratio; aOR, adjusted odds ratio; CI, confidence interval. Statistical significance is defined as P < 0.05. Data are presented as the percentage (frequency), OR (95% CI) or aOR (95% CI)*.

**Table 3:** Effects of age on pregnancy outcomes of women with or without PCOS.

**Variable**	**Non-PCOS**			**PCOS**		
	**<35 years (Group A)**	**≥35 years (Group C)**	***P*-value**	**<35 years (Group B)**	**≥35 years (Group D)**	***P*-value**
Clinical pregnancy rate, %	68.4 (8,191/11,974)	47.0 (2,044/4,346)		65.5 (1,491/2,277)	59.1 (178/301)	
OR (95% CI)	REF	0.410 (0.382–0.440)	0.000	REF	0.763 (0.597–0.975)	0.031
aOR (95% CI)	REF	0.651 (0.572–0.742)	0.000	REF	0.560 (0.426–0.736)	0.000
Miscarriage rate, %	5.7 (684/11,974)	8.0 (349/4,346)		7.9 (181/2,277)	9.0 (27/301)	
OR (95% CI)	REF	1.441 (1.261–1.648)	0.000	REF	1.141 (0.747–1.743)	0.541
aOR (95% CI)	REF	1.409 (1.231–1.614)	0.000	REF	1.503 (0.950–2.378)	0.082
Live birth rate, %	61.5 (7,362/11,974)	38.4 (1,668/4,346)		56.2 (1,280/2,277)	49.2 (148/301)	
OR (95% CI)	REF	0.390 (0.363–0.419)	0.000	REF	0.753 (0.592–0.958)	0.021
aOR (95% CI)	REF	0.641 (0.564–0.729)	0.000	REF	0.481 (0.363–0.637)	0.000
Singleton pregnancy rate, %	39.5 (4,728/11,974)	29.2 (1,267/4,346)		35.6 (811/2,277)	33.9 (102/301)	
OR (95% CI)	REF	0.631 (0.585–0.680)	0.000	REF	0.927 (0.719–1.194)	0.555
aOR (95% CI)	REF	0.816 (0.721–0.923)	0.001	REF	0.842 (0.649–1.092)	0.195
Preterm delivery, %	5.5 (269/4,728)	7.8 (99/1,267)		8.2 (67/811)	8.8 (9/102)	
OR (95% CI)	REF	1.204 (1.070–1.354)	0.002	REF	1.041 (0.736–1.473)	0.821
aOR (95% CI)	REF	1.183 (1.050–1.332)	0.006	REF	0.995 (0.701–1.413)	0.979
Large for gestational age, %	27.4 (1,295/4,728)	31.2 (395/1,267)		31.3 (254/811)	33.3 (34/102)	
OR (95% CI)	REF	1.135 (0.993–1.297)	0.064	REF	0.991 (0.646–1.521)	0.968
aOR (95% CI)	REF	1.060 (0.991–1.134)	0.087	REF	1.010 (0.814–1.253)	0.927
Small for gestational age, %	3.9 (186/4,728)	4.9 (62/1,267)		3.3 (27/811)	0.9 (1/102)	
OR (95% CI)	REF	1.206 (0.899–1.618)	0.212	REF	0.269 (0.036–1.995)	0.199
aOR (95% CI)	REF	1.108 (0.956–1.284)	0.172	REF	0.518 (0.190–1.413)	0.199
Multiple pregnancy rate, %	22.0 (2,634/11,974)	9.2 (401/4,346)		20.6 (469/2,277)	15.3 (46/301)	
OR (95% CI)	REF	0.360 (0.322–0.403)	0.000	REF	0.695 (0.500–0.967)	0.031
aOR (95% CI)	REF	0.503 (0.424–0.598)	0.000	REF	0.550 (0.385–0.786)	0.001
Preterm delivery, %	34.3 (1,807/5,268)	39.8 (319/802)		39.6 (371/938)	45.0 (41/92)	
OR (95% CI)	REF	1.266 (1.009–1.589)	0.042	REF	1.250 (0.652–2.397)	0.501
aOR (95% CI)	REF	1.111 (0.992–1.248)	0.069	REF	1.114 (0.803–1.544)	0.518
Large for gestational age, %	3.7 (196/5,268)	4.2 (34/802)		3.9 (37/938)	0 (0/92)	
OR (95% CI)	REF	1.134 (0.941–1.366)	0.188	REF	——	0.997
aOR (95% CI)	REF	1.141 (0.946–1.375)	0.167	REF	——	0.997
Small for gestational age, %	27.8 (1,252/5,268)	21.6 (173/802)		23.1 (217/938)	23.9 (22/92)	
OR (95% CI)	REF	1.008 (0.920–1.106)	0.861	REF	1.081 (0.835–1.399)	0.556
aOR (95% CI)	REF	1.002 (0.914–1.009)	0.963	REF	1.081 (0.835–1.399)	0.556
Cumulative live birth rate,%	72.6 (10,186/14,038)	45.3 (2,064/4,558)		69.3 (2,180/3,144)	60.6 (217/358)	
OR (95% CI)	REF	0.313 (0.291–0.344)	0.000	REF	0.678 (0.542–0.850)	0.001
aOR (95% CI)	REF	0.584 (0.523–0.652)	0.000	REF	0.443 (0.340–0.576)	0.000

*OR, odds ratio; aOR, adjusted odds ratio; CI, confidence interval. Statistical significance is defined as P < 0.05*.

*Data are presented as the percentage (frequency) and OR (95% CI) or aOR (95% CI)*.

**Table 4:** Cumulative live birth rate of women aged 35–40 years and those aged over 40 years.

**Variable**	**(Group C 1) non-PCOS ≥35 and <40 years**	**(Group D 1) PCOS ≥35 and <40 years**	**(Group C 2) non-PCOS ≥40 years**	**(Group D 2) PCOS ≥40 years**
Total number of IVF cycles	3,311	316	1,247	42
Age (y)^a,c,d^	36.70 ± 1.38	36.37 ± 1.30	41.89 ± 1.88	38.04 ± 2.76
BMI (kg/m^2^)^a,b,c^	22.98 ± 2.86	24.60 ± 3.38	23.59 ± 5.38	25.89 ± 3.74
Gn dosage (IU)^a,c^	3036.27 ± 948.03	2665.05 ± 1059.79	3431.98 ± 942.80	3065.48 ± 1096.04
Peak E2^a,b,c^	3665.20 ± 2334.81	4446.93 ± 2803.22	2652.97 ± 1973.00	3756.70 ± 2178.94
No. of retrieved oocytes^a,b,c^	9.16 ± 5.33	12.35 ± 6.74	6.85 ± 4.70	11.12 ± 6.23
No. of available embryos^a,b,c^	4.34 ± 2.72	5.91 ± 3.93	3.36 ± 2.30	5.33 ± 3.35
Fresh embryo ET cycles, %^a,b,c^	94.6 (3,128)	83.5 (264)	97.4 (1,215)	88.1 (37)
Implantation rate, %^a,b,c^	34.0 (2,197/6,456)	38.0 (212/558)	15.7 (363/2,311)	27.8 (22/79)
Clinical pregnancy rate, %^b,c^	55.2 (1,712)	60.2 (159)	26.1 (317)	51.4 (19)
Miscarriage rate, %	12.4 (139)	14.1 (14)	9.4 (55)	6.3 (1)
Live birth rate, %^b,c^	46.6 (1,457)	50.0 (132)	18.2 (221)	43.2 (16)
Cumulative live birth rate(CLBR),%^b,c^	54.2 (1,796)	62.0 (196)	21.5 (268)	50.0 (21)

“*a,” “b,” “c,” and “d” mean that significant differences were detected between groups A and B, C and D, A and C, and B and D, respectively. Statistical significance is defined as P < 0.05. Data are presented as the mean ± SD or the percentage (frequency). In the comparisons of the CLBRs between the four groups, adjustments were made for confounders*.

Among women with or without PCOS, older patients exhibited a lower clinical pregnancy rate [group C vs. A, aOR: 0.651 (0.572–0.742); group D vs. B, aOR: 0.560 (0.426–0.736); all *P* < 0.05] and LBR [group C vs. A, aOR: 0.641 (0.564–0.729); group D vs. B, aOR: 0.481 (0.363–0.637); all *P* < 0.05] than their younger counterparts. Similarly, among the non-PCOS patients, a significantly higher clinical pregnancy rate (55.2 vs. 26.1%, *P* < 0.05) and LBR (46.6 vs. 18.2%, *P* < 0.05) were observed in group C1 than in group C2. However, among the PCOS patients, the clinical pregnancy rates and LBRs of group D1 and group D2 were comparable (*P* = 0.263 and 0.385, respectively). Additionally, among the patients aged 35–40 years, no significant differences in the clinical pregnancy rate or LBR were observed between group C1 and D1 (*P* = 0.254 and 0.540, respectively). Among the patients over 40 years old, the PCOS patients (group D2) showed significant advantages in both the clinical pregnancy rate (51.4 vs. 26.1%, *P* < 0.05) and LBR (43.2 vs. 18.2%, *P* < 0.05) over the non-PCOS patients (group C2; Table 4).

### Fetal/Neonatal Outcomes of Fresh Embryo Transfer Cycles

Group B showed a significantly lower singleton pregnancy rate than group A [aOR: 0.877 (0.777–0.960); *P* < 0.05], but no significant difference was detected in the multiple pregnancy rate between these two groups (*P* > 0.05). In these singleton pregnancy cases, the preterm delivery rate was higher in group B than in group A [aOR: 1.419 (1.067–1.888); *P* < 0.05], while this situation was not observed for multiple pregnancy. In both singleton and multiple pregnancy cases, no significant difference was detected in the LGA and SGA rates between the two groups (all *P* > 0.05). The multiple pregnancy rate was higher in group D than in group C [aOR: 1.413 (1.006–1.983); *P* < 0.05], while the singleton pregnancy, preterm delivery, LGA, and SGA rates were comparable between group D and C (all *P* > 0.05; Table 2).

The multiple pregnancy rate [group C vs. A, aOR: 0.503 (0.424–0.598); group D vs. B, aOR: 0.550 (0.385–0.786); all *P* < 0.05] was lower in older patients with or without PCOS than in their younger counterparts. Among non-PCOS patients, group C had a lower singleton pregnancy rate [aOR: 0.816 (0.721–0.923); *P* < 0.05] than group A, while among PCOS patients, the singleton pregnancy rate was comparable between the different age groups (*P* > 0.05). In these singleton pregnancy cases, group C showed a significantly higher preterm delivery rate than group A [aOR: 1.183 (1.050–1.332); *P* < 0.05], while in multiple pregnancy cases, the preterm delivery rate was comparable among the different groups (all *P* > 0.05). The LGA and SGA rates were comparable among the different age groups of PCOS patients and non-PCOS patients (all *P* > 0.05; Table 3).

### Comparison of CLBRs Among Groups (Tables 2, 3)

Confounders associated with CLBR in the single-factor logistic regression analysis, including age, BMI, total Gn, and the number of oocytes retrieved, were adjusted in the multiple regression analyses.

The CLBR was statistically higher in the group of non-PCOS patients aged less than 35 years than in the group of non-PCOS patients over 35 years old (72.6 vs. 45.3%, *P* < 0.05; Table 3). Similarly, the non-PCOS patients aged 35–40 years showed a significantly higher CLBR than the non-PCOS patients over 40 years old (54.2 vs. 21.5%, *P* < 0.05; Table 4).

Among women over 35 years of age, the PCOS patients exhibited a higher CLBR than the non-PCOS patients (60.6 vs. 45.3%, *P* < 0.05; Table 2). Similarly, the CLBR was higher in the group of PCOS patients over 40 years old than in the group of non-PCOS patients over 40 years old (43.2 vs. 18.2%, *P* < 0.05). However, the CLBRs were comparable between the PCOS patients and non-PCOS patients aged 35–40 years (62.0 vs. 54.2%, *P* = 0.219); Significant differences were found in the CLBRs of the PCOS patients over 35 years old and their younger counterparts (60.6 vs. 69.3%, *P* < 0.05; Table 3). However, no significant difference in the CLBR was detected between the PCOS patients aged 35–40 years and those aged over 40 years (62.0 vs. 50.0%, *P* = 0.112; Table 4).

### Age-Related Changes in Treatment Outcomes (Figure 2)

In the non-PCOS group, age was negatively associated with a reduced number of retrieved oocytes (slope −0.049, SE: 0.009, *P* < 0.05) and implantation rate (slope −0.014, SE: 0.001, *P* < 0.05). In the PCOS group, no association was detected between the number of retrieved oocytes and age (slope −0.003, SE: 0.026, *P* > 0.05), while the implantation rate was negatively associated with age (slope −0.009, SE: 0.002, *P* <0.05). The slopes of the regression lines associated with the oocyte number and implantation rate were all significantly different between the two groups (*P* < 0.05).

**Figure 2:**
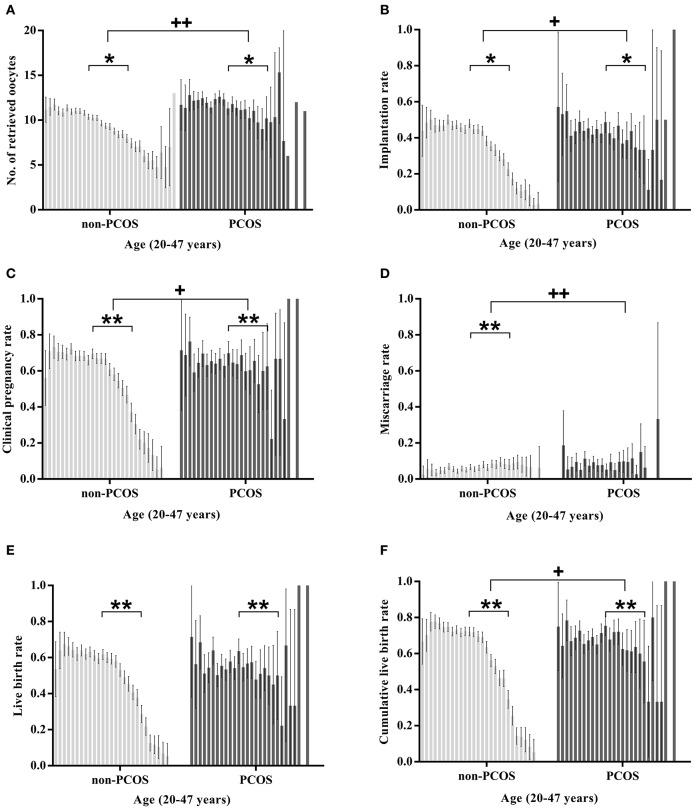
Age-related changes in the treatment outcomes of women aged 20–47 years with or without PCOS. The treatment outcomes in **(A–E)** are calculated per fresh embryo transfer cycle; The cumulative live birth rates in **(F)** are calculated in a completed single stimulation cycle. “+” Slopes are significantly different between the two groups; “*” there is a significant association between age and the statistic (“*” or “+”*P* < 0.05; “**” or “++” *P* < 0.001).

In the PCOS group, both the clinical pregnancy rate and LBR were negatively associated with age (coefficient: −0.043 and −0.046 respectively, all *P* < 0.05), and no significant association was observed between age and the miscarriage rate (coefficient: −0.029, *P* > 0.05). In the non-PCOS group, the clinical pregnancy rate, LBR and miscarriage rate were all significantly associated with age (coefficient: −0.076, −0.081, and 0.032, respectively, all *P* < 0.05). The CLBRs of both the PCOS group and non-PCOS group were negatively associated with age (coefficient: −0.048 and −0.098, all *P* < 0.05). The change in the clinical pregnancy rate, miscarriage rate, and CLBR by age (20–47 years) between the two groups was significantly different (all *P* < 0.05), while the changes in the LBRs of the two groups were comparable (*P* > 0.05).

### Cumulative Live Birth Rate-Related Variables (BMI, Age, and the Number of Retrieved Oocytes; Figure 3)

Logistic analyses showed that in younger non-PCOS patients (Group A), age [aOR: 0.982 (0.969–0.995); *P* < 0.05], BMI [aOR: 0.976 (0.964–0.989); *P* < 0.05], and the number of retrieved oocytes [aOR: 1.022 (1.015–1.029); *P* < 0.05] were correlated with CLBR before and after adjusting for relevant confounders. However, in younger PCOS patients (Group B), only BMI [aOR: 0.961 (0.939–0.985); *P* < 0.05] was negatively correlated with CLBR. In older non-PCOS patients (Group C) and older PCOS patients (Group D), age [aOR: 0.769 (0.748–0.790), aOR: 0.891 (0.803–0.990); all *P* < 0.05] and the number of retrieved oocytes [aOR: 1.041 (1.027–1.054), aOR: 1.039 (1.002–1.078); all *P* < 0.05] were associated with CLBR; No significant associations were detected between BMI and CLBR in either Group C or Group D (*P* = 0.271 and 0.203, respectively).

**Figure 3:**
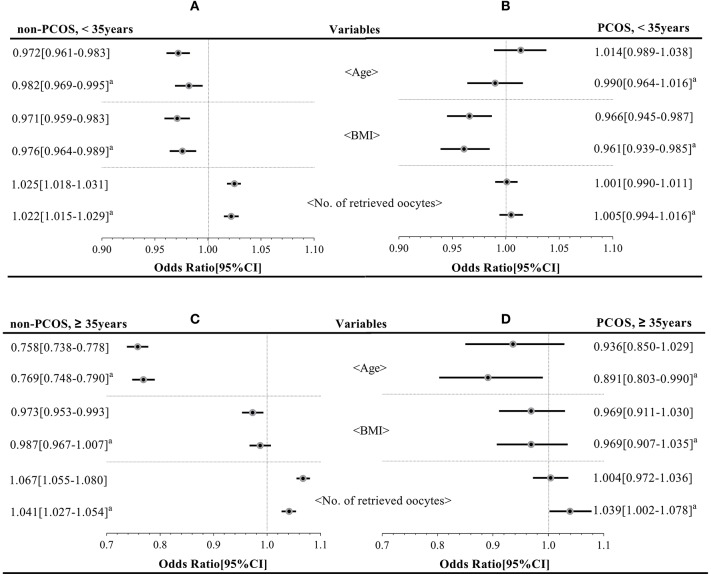
Key factors affecting the cumulative live birth rate of the four groups (group **A–D**). ^a^Means the OR with the 95% CI has been adjusted for age, BMI, total Gn, and the number of oocytes retrieved.

## Discussion

To evaluate the effects of increasing age on the pregnancy outcomes of PCOS patients, we retrospectively analyzed clinical data from 22,098 infertile women who underwent their first IVF-ET attempt and subsequent FET cycles. Age is closely related with female fertility, and therefore, considering the special endocrine characteristics of women with PCOS, the extent to which age affects this group requires further research. This study aimed to explore age-related pregnancy outcomes in women with PCOS. Interestingly, we found that after the age of 35 years, PCOS patients showed better pregnancy outcomes than age-matched normal populations. The decline in fertility occurred at a slower rate in PCOS patients than in normo-ovulatory women as age increased.

### Relationship Between Age and Reproductive Characteristics of PCOS

In our study, younger women (<35 years) with PCOS demonstrated an ~23% higher number of retrieved oocytes and ~18% more available embryos than aged-matched non-PCOS patients, while older (≥35 years) women with PCOS demonstrated an almost 43% higher number of retrieved oocytes and available embryos than aged-matched non-PCOS patients. The higher oocyte yield and greater number of available embryos may contribute to greater transplantation opportunities in subsequent FET cycles. This study mainly assesses how advanced age affects the pregnancy outcomes of women with PCOS in a whole IVF cycle, including the fresh ET cycle and subsequent FET cycles.

### Effects of Age on the Pregnancy Outcomes of Fresh Embryo Transfer Cycles

In our study, one to three embryos were transferred per cycle. One- or three-ETs mainly occurred among women over 35 years old who had fewer available embryos or were generally considered to have age-related poor fecundity. To exclude the effects of the number of transplanted embryos on pregnancy outcomes, the embryo implantation rates were calculated to detect early pregnancy outcomes in the different groups.

Among the women aged less than 35 years old, no significant differences were observed in the implantation and clinical pregnancy rates between PCOS patients and non-PCOS patients after adjusting for relevant confounders; however the LBR was higher for the non-PCOS patients than for the PCOS patients. With increasing age, the implantation rate and pregnancy rate in PCOS patients and non-PCOS patients declined at a different rate. Thus, among the women aged 35 years, the PCOS patients showed a significant advantage in embryo implantation and clinical pregnancy over the non-PCOS patients. Although the decline rates of LBR among patients with and without PCOS were comparable, our results also suggested that the LBR of PCOS patients aged over 35 years was higher than that of same-aged non-PCOS patients; the pregnancy advantages for PCOS patients were significant, especially for patients over 40 years old. A previous study conducted by Kalra et al. indicated that after the age of 40 years, women with PCOS do not present any advantages in terms of embryo implantation, clinical pregnancy, or LBR over women with tubal factor infertility despite having an ~35% higher oocyte yield (12). The following reasons may explain this inconsistency with our results: we calculated and analyzed the clinical pregnancy rate and LBR per ET cycle but not per each cycle started, and we adjusted for BMI in our analysis, which was not performed in the previous study.

A few studies have suggested that BMI is a major initiator of the high miscarriage rate in women with PCOS (18, 19). A large cohort study has shown a higher miscarriage rate in PCOS patients aged 28–33 years than in age-matched normo-ovulatory women. However, in that study, no adjustments were made for the effect of BMI on the miscarriage rate (20). In our study, after adjusting for BMI, the miscarriage rate was higher in young PCOS patients (<35 years) than in young non-PCOS patients. Interestingly, with increasing age, the miscarriage rate in PCOS patients showed no significant change, while the miscarriage rate in non-PCOS patients increased significantly. Thus, no significant change was detected in the miscarriage rate between older PCOS and non-PCOS patients.

### Effects of Age on the Cumulative Live Birth Rate

Compared with those of the age-matched non-PCOS patients in the different age groups, namely, <35, 35–40, and ≥40 years, the CLBRs of the PCOS patients were lower, comparable, and higher, respectively; this is consistent with our finding that the declining slopes of CLBR with age in patients with and without PCOS were significantly different. A previous study indicates that the cumulative LBR continuously increases with the number of oocytes retrieved (21). Thus, the higher CLBR of PCOS patients over 40 years old may also be due to an almost 62.3% higher number of retrieved oocytes from the PCOS patients than from age-matched non-PCOS patients. Another retrospective study also indicated that a higher number of oocytes from older PCOS patients (≥40 years) can result in an improved CLBR compared with non-PCOS patients (22). However, only a small number of PCOS patients were included in this study, and the CLBR of younger PCOS patients was not examined (22). The implantation rate, clinical pregnancy rate, LBR, and CLBR all showed significant declines among PCOS patients aged 20–47 years; however, when pregnancy outcomes of PCOS patients aged 35–40 years were compared to those of PCOS patients aged over 40 years, better pregnancy outcomes trends were observed in the younger group, but statistical significance was not reached. This may due to the limited number of PCOS patients aged over 40 years.

Among PCOS patients, BMI is the main factor that influences the CLBR of younger age groups. However, in older age groups, the critical role of BMI is replaced by the age-related decline in fertility. Previous research suggests that the age-related decline in fertility has a greater influence on CLBR than BMI in older women (23), which is consistent with our findings. Weight loss before IVF is widely recommended for women who are overweight or obese to improve underlying adverse pregnancy outcomes (24); however, the benefits of weight loss before ART interventions have been questioned in some clinical trials (25, 26), and undergoing fertility treatment before losing weight is recommended (27). It seems that achieving a lower BMI prior to IVF may be a good choice for younger PCOS patients. However, taking time to lose weight before IVF treatment may not be effective in improving the CLBR of older PCOS patients.

### Effects of Age on the Fetal/Neonatal Outcomes Associated With Fresh Embryo Transfer Cycles in PCOS Patients

Assisted reproductive technology is usually accompanied by multiple pregnancies. Additionally, preterm birth (PTB) and lower neonatal birth weight are the main reasons for morbidity and mortality in multiple pregnancies. In the present study, compared with younger non-PCOS patients (<35 years), both younger PCOS patients and older non-PCOS patients (≥35 years) had a higher PTB rate of singleton pregnancy cases after adjusting for BMI. This finding may suggest that the presence of maternal, fetal, or placental abnormities resulting from advanced age or pathological characteristics of PCOS can lead to induced or spontaneous preterm delivery. A previous study by Qin et al. suggested that PCOS patients exhibit a higher PTB rate, which is in accordance with our results (28). Cervical insufficiency is frequently detected in the PCOS population, compared with normo-ovulatory women, but the underlying mechanism is not clear (29). Conversely, in our multiple pregnancy cases, no significant differences in the PTB rate were detected among the four groups.

A meta-analysis of studies involving 20,965 women pregnant with twins showed that women with PCOS had a higher PTB rate and low newborn birth weight than women without PCOS. However, no significant differences in newborn birth weight were observed after adjusting for BMI and gestational age (30). We compared the SGA and LGA rates in both singleton and multiple pregnancy cases in different groups. After adjusting for maternal age, BMI, base FSH, LH, T, peak E2 level, number of transplanted embryos, and neonatal gender, no significant differences were detected in the SGA or LGA rate among the different age groups. However, our findings are in contrast to other previous studies suggesting that hyperandrogenism and/or insulin resistance may contribute to histological changes in local microvascular structures in the trophoblast and placental tissue of women with PCOS, which can affect maternal-fetal oxygen and nutrient transfer that is essential for fetal growth during pregnancy (31, 32). To date, the relationship between PCOS and intrauterine growth of the fetus remains controversial. Some other studies have shown no significant difference in the SGA rate between PCOS and non-PCOS patients (33, 34), consistent with our results. Another later meta-analysis found that PCOS patients showed almost a two-fold higher risk of SGA but no difference in LGA risk compared with normo-ovulatory patients (35).

### Strengths and Limitations

This is a comparative, large-sample-sized study based on the CCRM/EMRCD, which provides reliable medical records. The CLBR, which is an estimate of the final ART success rate, appears to be a much better indicator of the quality and success of IVF programs for this specific group of patients, and the interaction between advanced reproductive age and distinct reproductive characteristics of PCOS in terms of the CLBR is seldom studied.

Nevertheless, our study has some limitations. First, this study had a retrospectively design. The data on the use of metformin before or during ovarian hyperstimulation were not recorded. Second, the included patients all underwent the GnRH agonist controlled ovarian hyperstimulation protocol, and only cleavage-stage embryo transfers in fresh cycles were analyzed, which may reduce the generalizability of our results. Overall, this result should be interpreted with caution. Third, only a relatively small number of patients of advanced age were included, especially in the group of older PCOS patients. We cannot exclude that the different treatment outcomes between the younger and older patients are the result of this limited sample size. Finally, due to incomplete follow-ups, fetal/neonatal outcomes in FET cycles were not analyzed in our study; only the fetal/neonatal outcomes associated with fresh cycles were compared among the different groups.

## Conclusion

In conclusion, unlike non-PCOS patients, whose reproductive capacity generally declines sharply after 35 years of age, PCOS patients have a slower decline in fecundity associated with IVF, and advantage in fecundity can be expected in PCOS patients over 40 years old. However, advanced age still plays a critical role in the ultimate success of a single stimulation cycle in PCOS patients. Controlling BMI before IVF treatment is beneficial for younger PCOS patients (<35 years), but for older PCOS patients (≥35 years), losing weight before IVF treatment may not help in achieving final treatment success. Thus, initiating infertility treatment immediately is essential. Additionally, no significant detrimental effect associated with the fetal/neonatal outcomes of fresh IVF cycles was detected in older PCOS patients (≥35 years), but further research is still needed to evaluate the health outcomes of the offspring over the long term.

## Data Availability Statement

The datasets generated for this study are available on request to the corresponding author.

## Ethics Statement

The studies involving human participants were reviewed and approved by The Ethics Committee of the First Affiliated Hospital of Zhengzhou University. The patients/participants provided their written informed consent to participate in this study.

## Author Contributions

JL and XL: study design, analysis and interpretation of data, and drafting of the manuscript. FW, HK, and SD: data collection. LH and FZ: assessed the article. YG: study concept and revise of article. All authors approved the final article.

### Conflict of Interest

The authors declare that the research was conducted in the absence of any commercial or financial relationships that could be construed as a potential conflict of interest.
